# An Assessment of the Genetic Parameters of Boars’ Reproductive Traits

**DOI:** 10.3390/genes14112003

**Published:** 2023-10-27

**Authors:** Emil Krupa, Zuzana Krupová, Eliška Žáková, Jiří Bauer, Nina Moravčíková, Irena Vrtková

**Affiliations:** 1 Institute of Animal Science, 10400 Prague, Czech Republic; krupova.zuzana@vuzv.cz (Z.K.); zakova.eliska@vuzv.cz (E.Ž.); 2 Czech-Moravian Breeders Association, 25209 Hradistko, Czech Republic; bauer@plemdat.cz; 3 Institute of Nutrition and Genomics, Slovak University of Agriculture, 94901 Nitra, Slovakia; nina.moravcikova@uniag.sk; 4 Department of Morphology, Physiology and Animal Genetics, Mendel University, Zemedelska 1, 61300 Brno, Czech Republic; irena.vrtkova@mendelu.cz

**Keywords:** pig, sperm characteristics, libido, heritability, ssGBLUP

## Abstract

The aim of this study was to estimate genetic parameters for the reproductive traits of boars based on single-nucleotide polymorphism data. A total of 109,836 semen samples from 2249 boars were collected between 2010 and 2022. Five basic traits were assessed: sperm volume, sperm concentration, motility, number of abnormal sperm, and, for the first time for the local population, libido. In addition, two derived traits were assessed: total sperm count and number of functional sperm. Genetic parameters were estimated using the single-step genomic best linear unbiased prediction method (ssGBLUP). Dam and sire breeds were evaluated separately. The five basic traits were evaluated using five-trait models, while the two derived traits were evaluated using single-trait models. The heritability coefficients had lower values for all sperm quality traits with both methods. For the basic traits, the heritability ranged from 0.099 to 0.342. The greatest difference between dam and sire breeds was observed for the heritability of the sperm concentration trait (0.099 and 0.271, respectively). The heritability of the libido trait was twice as high for boars of sire breeds as it was for boars of dam breeds. The genetic parameters estimated with ssGBLUP can be used in routine genetic evaluations to improve the pig breeding process.

## 1. Introduction

The insemination of sows has become a very useful technique that is widely used in swine production. In addition to the classical productive and reproductive traits of sows and boars, their sperm characteristics and libido also play an important role. An emphasis on improved sperm quality and quantity can be beneficial in terms of the economics of insemination stations [1], as well as in the context of the profitability of integrated swine-production systems [2]. Therefore, the improvement of such functional traits could influence the swine sector with regard to its complexity.

Sperm characteristics and libido form the mainstay of the reproductive evaluation of breeding boars. According to Knecht et al. [3], the main parameters responsible for boar AI culling include a low semen value (23.7%) and reduced demand for semen from the given boar (22.5%). However, leg problems resulted in a 14.9% culling rate, and a low or lack of libido represented only a 9.3% culling rate. It was reported that almost half of the selected young breeding boars were culled due to low-sperm-quality parameters [4]. Similarly, the authors of [5] identified genetic improvements, poor semen quality, and foot and leg issues as the major reasons for boar replacement. Therefore, the reproductive abilities of boars should be considered together with their productive and other functional traits when constructing comprehensive selection criteria for breeding boars. The effect of inbreeding depression on the genetic evaluation of various reproductive traits in boars of different populations has also been studied in the literature [6,7]. To achieve the highest possible efficiency in the production of insemination doses, pressure is put on the whole process. However, this is largely dependent on the reproductive cycle of the boar. The sexual maturity in domestic pigs occurs between seven and nine months under the influence of genetic, social, and environmental factors [8]. There are many papers oriented on analyses of factors affecting the reproduction cycle of boars [5,9,10]. 

The estimation of the breeding values of sperm characteristics and libido is intended to reflect the genetic potential of animals through these traits. The estimated parameters can provide useful information for the establishment of a comprehensive breeding scheme in purebred sire [11,12] and dam [10,12,13] breeds and in various pig lines, i.e., crossbreeds [14,15]. Studies focused on genetic evaluation have varied in their range and in the way semen production and quality are expressed. The traits that are measured directly are represented by the semen volume, the sperm concentration, which is log-transformed [14] and expressed as a percentage [16], sperm motility (e.g., in the last two cited studies), and sperm progressive motility [17]. Further, the total number [17] and percentage of abnormal sperm [10,14], the proportion of morphologically normal sperm (an alternative to [11]), and various characteristics that define abnormalities in sperm morphology [18] have been examined. The traits derived from direct measurements include the total number of sperm in the ejaculate [10,11], the number of functional spermatozoa, and the number of insemination doses [15]. Studies evaluating the libido (expressing the willingness of boars to mount) are rare (e.g., [12]); they indicated genetic parameters that were variable among dam and sire breeds and evaluated their traits (reproductive, growth, locomotion, exterior, and feeding traits).

A general task at present is the implementation of molecular information into routine breeding processes to enhance the genetic progress of such traits. The detection and application of novel candidate genes for sperm characteristics through marker-assisted selection is generally considered [13] to provide information for boar selection at an earlier age. Molecular techniques open the possibility for a deeper study and understanding of the genetic mechanisms, pathways, and complex processes underlying the traits of semen production and quality (e.g., [11]), even when considering the transcriptome and the traits’ seasonal variability in animals [19]. The authors of [20] reported that the use of genomic data was successful enough to achieve a 50% greater genetic gain in traits of interest. 

Even though semen traits have not yet taken a place among the breeding goals for the Czech pig population [21], they have been partially prioritized by local breeders [22], and their designation as local breeding goals (especially for sire breeds) is desired with respect to their direct economic importance [2]. A previous genetic evaluation [10] also pointed out that AI centers should place an optimal emphasis on the associated breeding values. Therefore, the routine estimation of the breeding values of semen traits has been provided in the local pig population for more than a decade (based mainly on [10,15]). Recently, a simultaneous assessment of SNP data and pedigree data from an endangered Czech pig breed was carried out in a pilot study [23] based on the ongoing genotyping of the local pig population. The aim of the present work was to provide a comprehensive evaluation of the genetic parameters and genomic estimates of semen traits in the commercial Czech pig population to be further applied in animal selection. The estimates for the libido trait were provided for this local pig population for the first time.

## 2. Materials and Methods

### 2.1. Phenotypes

The data, which were provided by the Czech Pig Breeders’ Association, were routinely collected from twelve insemination stations between 2010 and 2022 for genetic evaluation as part of CzePig (the Czech national pig breeding program). Altogether, 109,836 semen samples were collected within the evaluation period. The data included the ejaculates and sexual behavior of boars from two dam breeds (Czech Large White—CLW, Czech Landrace—CL) and three sire breeds (Duroc—DC, Sire Line Large White—SLLW, Pietrain—PN). The dam breeds are used in the Czech national breeding scheme in the maternal position as they are carriers of excellent female reproductive traits such as numbers of piglets born and weaned, and milk yield. On the other hand, sire breeds are characterized by excellent meat-quality parameters. The design of the study was based on a routine genetic evaluation in the Czech national pig breeding program. In this scheme, a joined genetic evaluation for two dam breeds is used, and separately a joined evaluation for three sire breeds is applied. The reason for this is, on the one hand, the relatively close similarity of the maternal breeds bred in the Czech Republic and, on the other hand, the small population of individual sire breeds. Therefore, two groups (D and S) were formed from the original dataset for animals of dam and sire breeds, respectively. 

A total of seven traits describing the reproduction of boars were analyzed. The following were assumed as the basic traits:

- Semen volume (SV)—measured in milliliters per ejaculate;
- Sperm concentration (SC)—measured as 10^3^ cells per mm^3^;
- Motility (MO)—measured as the proportion of sperm moving in a straight line (as a percentage);
- Abnormal sperm (AB)—measured as the proportion of deformed or otherwise defective sperm;
- Libido (LI)—expressing the willingness to mount a phantom sow; this was measured subjectively on a five-point scale, where one was the worst and five was the best expression of libido (1 = unsatisfactory, 2 = below average, 3 = average, 4 = very good, 5 = excellent); 0 = did not mount.

Two other traits were derived from the basic traits:

The total number of sperm per boar collection (TNS) in 10^9^ sperm:

TNS = SV × SC/1000.(1)

The number of functional sperm per boar collection (NFS) in 10^9^ sperm:

NFS = TNS × (MO/100) × (1 − AB/100).(2)

In total, reproductive traits were available for 2249 boars. The analyses of variances for the evaluated traits and factors were performed in R-project [24]. Trait observations that did not meet the minimal and maximal values were excluded. In addition, boars with fewer than three sperm collections were excluded. The main factors affecting the evaluated traits and explaining a major proportion of the variability were the age of the boar at collection, the year and month of collection, the interval between two consecutive collections, and the insemination station. Collections obtained before the age of 6 months and after the age of 58 months were excluded. An interval of 3 days was the minimum threshold for the inclusion of traits in analyses. The effect of the age of the boar was adjusted into classes. Monthly intervals were assumed up to an age of 30 months. Bimonthly intervals were formed for ages from 31 to 40 months. Classes were formed for the rest of the ages: 41–43, 44–47, 48–52, and 53–58 months. Similarly, classes were formed for the effect of the interval between two collections. A one-day class was formed for intervals up to 11 days. For longer intervals, the following classes were formed: 12–13, 14–15, 16–20, 21–30, and more than 30 days. Two insemination stations were excluded due to their low number of observations. Boars that reached a minimum age of 7 months (sexual maturity) were quarantined at the insemination station, and had undergone test collections which were included in further analyses. The test collections were not included in the analyses. Boars were fed a mixture in accordance with the recommendations for feeding breeding boars, considering the growth needs of young boars and maintaining the good condition of mature boars. The mixture was optimized for selenium and mineral content. Finally, 56,852 sperm collections from 1209 boars were divided into two groups (D and S) and used for further analyses.

### 2.2. Genotypes

Genome-wide data were available for 1763 individuals (1030 CLW animals, 401 CL animals, 98 DC animals, 89 SLLW animals, and 145 PN animals) born between 2005 and 2022. Bristles, frozen blood samples, ear tissue samples, and used insemination dose samples were employed as the sources of DNA. All DNA analyses were performed at the laboratory of genetics of the Czech–Moravian Breeders’ Corporation (Hradištko, Czech Republic). DNA was isolated using the magnetic bead method and the silicate column method for the bristles and insemination samples, respectively. DNA from the blood samples was isolated using the GeneAll Eugene DNA micro isolation kit (GeneAll Biotechnology, Seoul, Republic of Korea). First, an evaluation of the quality of the DNA isolates (standard measurement of concentration and purity and other control steps based on spectrophotometry, multiplex PCR, and electrophoresis) was performed. The selected DNA isolates were applied to two different arrays. The PorcineSNP60 v2 BeadChip array (61,565 SNPs) and the GGP Porcine 50 k array (50,697 SNPs) were used for 658 and 1105 animals, respectively. The average call rates were 0.99, 0.97, and 0.94 for the bristles, blood samples, and insemination samples, respectively. Animals with a call rate higher than 0.89 were used in the subsequent analyses. Only the SNPs that were present on both arrays were considered for further evaluation (32,897 SNPs). Only SNPs mapped on autosomal chromosomes were considered in this study. PLINK v. 1.9 software [25] was used for quality control and subsequent analyses. SNPs with a call rate of >0.90, a *p*-value of Hardy–Weinberg equilibrium of >0.0001, and a minor allele frequency (MAF) of >0.05 were included in the SNP analyses. After the quality-control analysis of all SNPs, the data from 1681 animals containing 32,345 SNPs were used for further investigation. All of the genotypes were stored in TheSNPpit database [26], a performance database system for managing large-scale SNP data.

### 2.3. Statistical Models

The genetic parameters were estimated with the single-step genomic best linear unbiased prediction method (ssGBLUP) using the phenotypes, pedigree information, and genotypes. Two main datasets were established. The first dataset (D) consisted of information from dam breeds (CLW, CL), and the second dataset (S) contained information from sire breeds (DC, SLLW, PN). The basic boar reproductive traits were evaluated together using a five-trait animal model. Single-trait models were applied for the derived sperm-quality traits (TNS, NFS). For each method and dataset, a statistical model was formed as follows:

Y_ijklmno_ = ym_i_ + age_j_ + int_k_ + sy_l_ + breed_m_ + p_n_ +a_n_ + e_ijklmno_, (3)

where y represents the evaluated boars’ reproductive traits measured in the oth sperm collection of the nth boar of the mth breed with the lth effect of the insemination station and year of collection, the kth class effect of the interval between subsequent collections, the jth class effect of the boar’s age at collection, and the ith year–month effect. p_n_ represents a permanent environmental effect on the boar; a_n_ is an additive genetic effect on the boar; and e_ijklmno_ represents a residual effect. The genetic parameters were estimated using the VCE method, and airemlf90 was run for the estimation of the variance components as implemented in [27]. The calculation was terminated when it converged to 10^−12^ or reached a convergence criterion (at a maximum of 2000 iterations) (the average number of iterations needed to meet the convergence criterion was less than 200 across all estimations). The standard errors of estimates were assessed with an approximation method described in the tutorial for the BLUPF90 program [28]. Breeding values were estimated with the BLUPF90+ program. The accuracy of the breeding values was estimated with the ACC program [29]. The calculations of the Spearman’s rank correlation coefficients were performed using R-project package corrr [24].

## 3. Results

### 3.1. Analyses of Phenotypes

Collection was started at 313 days of age on average for boars of dam breeds; 54.79 collections were made over a period of 412 days, and the boars produced sperm in 1.15 insemination stations on average. The sire-breed boars were characterized as males that started production at an average age of 338 days and stayed at an insemination station for 489.31 days on average. During this period, an average of 65.00 collections were made, and 1.10 inseminations were performed. The greatest number of collections took place in the months of March and April for both groups of boars. The shortest intervals between collections were also recorded in March and April, again for both groups of boars. Basic information on the evaluated datasets and traits is summarized in Table 1. Differences in mean values between datasets were evident for almost all traits. The dam-breed boars had a greater sperm volume and slightly higher motility and proportion of abnormal sperm. On the other hand, the boars of sire breeds had a higher sperm concentration and, as a result, achieved a higher total sperm number and number of functional sperm. The average value for libido was the same for both datasets. The average lengths of stay at an insemination station were 15.6 months and 19.0 months for boars of dam and sire breeds, respectively.

The average monthly trends for the evaluated traits in this recent period (2010–2022) are summarized in Supplementary Figure S1. Over the years, there was a decrease in SV from January to May in both datasets (D—dam and S—sire breeds), followed by a period of increasing SV until December. Similar trends were also observed for TNS and NFS. The libido of the boars of sire breeds reached a higher value during the year than that of the boars of dam breeds. Differences between the boars of dam breeds and the rest of the boars were also noted in the trends of libido during the year.

Supplementary Figure S2 shows the effect of the boar’s age at ejaculation (in months) on each evaluated trait. The sperm volume increased in all boars until the age of approximately 41 months. After this age, there was a decrease in semen volume. The “ideal” age of a boar for sperm production was considered to be in the interval between 18 and 28 months in terms of most of the observed traits. There was a noticeable increase in the value of only the AB trait for the boars of dam breeds. The optimal interval between two consecutive ejaculations appeared to be six days. A shorter interval generally had a negative effect on the traits that were evaluated. While prolonging the interval led to a partial improvement of some traits (SC, TNS), it also led to a deterioration of sperm motility and libido (confirmed by local breeders through personal communication). The effects of the interval between subsequent ejaculations are summarized in Supplementary Figure S3.

Table 2 and Table 3 show the variance ratios calculated for the factors affecting the evaluated traits. The proportion of explained variability captured by the model varied among the traits and datasets and could be simply derived from the residual variability. The highest variance was achieved for the motility trait (MO) in both datasets (higher than 60%). However, the lowest values were observed for AB in sire boars (less than 15%). Generally, the available effects captured greater proportions of variability in dam boars (except for SV) than in the sire boars. The greatest part of the variability was explained by the combined effects of the insemination station and the year of sperm collection. In addition, it must be mentioned that all effects in each model were highly statistically significant.

### 3.2. Genetic and Genomic Parameters

The coefficients of heritability (on the diagonal) and the genetic (above the diagonal) and phenotypic (below the diagonal) correlations between the evaluated traits in the five-trait ssGBLUP animal models are summarized in Table 4 and Table 5 for the dam and sire breeds, respectively. The genetic parameters for the derived reproductive traits (i.e., TNS and NFS) are shown in Table 6.

The heritability coefficients had lower values for all sperm-quality traits. For the basic traits, the heritability ranged from 0.099 (for SC in dam-breed boars) to 0.280 (for SV in dam-breed boars). Some of the heritability coefficients found for the dam and sire breeds even showed significant differences between the populations. The greatest difference was observed for the SC trait; for dam breeds, it reached a value of 0.099, whereas for sire breeds, it was 0.276. Slightly higher values of the heritability coefficients were obtained in the dam breeds for SV, MO, and AB. The opposite trend was observed for the LI trait (higher heritability in sire breeds). The coefficient of heritability for libido was twice as high for boars of the sire breeds as it was for boars of the dam breeds.

Similarly, for the derived traits, which were evaluated separately with single-trait models, lower heritability coefficients were obtained. There were basically no differences when comparing the heritability values between the evaluated populations. Although a small difference was observed for TNS, where the heritability value for the dam breeds was 0.149, for the sire boar population it was 0.123. The heritability coefficient for NFS reached values of 0.153 and 0.152 for the dam and sire breeds, respectively. The variance caused by the permanent effects of derived traits reached values from 0.235 (for TNS in sire-breed boars) to 0.287 (for TNS in dam-breed boars).

The genetic correlations among the sperm-quality traits ranged widely from moderately negative to moderately positive. According to the results, libido did not seem to have a negligible genetic influence on some of the evaluated reproductive traits. Moderately positive correlations were found among libido, SV, and MO, whereas a moderately negative relationship was detected with AB when boars of dam breeds were evaluated. A weak negative genetic relationship was observed between LI and SC.

**Figure 1:**
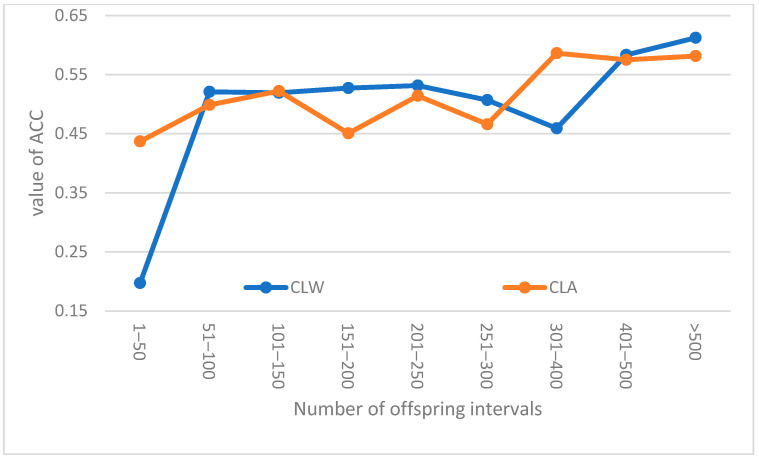
Trends of the estimated accuracies for the sperm volume in boars of the CLW and CLA breeds depending on their number of offspring.

Table 7 contains the basic statistical parameters for the breeding values and their accuracies (in parentheses) expressed for all evaluated breeding boars and their basic sperm quality traits. Further, Figure 1 presents some trends of the estimated accuracies of the sperm-volume values for breeding boars of the CLA and CLW breeds in their dependence on their numbers of offspring. A positive trend in accuracy could be seen in both breeds. A number of offspring greater than 301 did not affect the average accuracy of the breeding value for the CLA breed, whereas in the CLW breed, the average accuracy increased continuously.

## 4. Discussion

In our study, unlike many with a similar focus [11,12,18,30], we estimated the genetic parameters for multiple breeds of the same group (dam and sire breeds) in a single estimate. From our previous study on the evaluation of productive or reproductive traits [31], as well as from a detailed economic evaluation of production systems [2] in the local pig population, it was found that there were no remarkable differences in the parameters achieved for individual dam breeds raised in the Czech Republic. The use of the breed’s effect sufficiently (and significantly) captured the different levels of variability in the two evaluated dam breeds. Moreover, for the sire breeds, the joint evaluation was a practical solution, especially considering the lower number of boars of sire breeds that were evaluated.

The heritability coefficients for the sperm characteristics found in the present study ranged from 0.099 to 0.342. Comparable results were attained by the authors of the studies summarized in Table 8. The statistical data presented there contain only estimates based on single- or multi-trait animal models when the type of breed could be identified. Wolf [32,33] analyzed the sperm characteristics of boars bred in the Czech Republic. These analyses were carried out on the same breeds as those in our current study, but they were conducted more than 20 years ago. There is no direct intersection of data between the previous and the current study (i.e., the authors of [33] used data from 1995 to 2008), but a partial influence through the offspring of the previously evaluated boars could be assumed. The coefficients of heritability presented in Wolf’s studies on dam breeds (Czech Large White and Czech Landrace) reached values ranging from 0.17 for SV to 0.37 for AB. Likewise, in the framework of [32], the heritability coefficients of the sperm characteristics estimated for dam and sire breeds reached similar values to those presented in the present study. A slight difference between the breed groups was found only in the heritability recorded for two semen traits (AB and SC).

The genetic potential of the evaluated sperm characteristics appeared to be similar for the sire and dam breeds. The heritability coefficients summarized in Table 8, together with the numbers of estimates and sources, showed minimal differences in the mean values between breed groups (except for the proportion of abnormal sperm), whereas some differences are visible when comparing values between studies. This corresponds to the different variabilities in the values in the selected studies. In addition, in their publication, the authors of [34] presented average values of the heritability of semen quality traits regardless of the breed group. The average heritability coefficient was 0.19 for both SV and SC. MO and AB had average values of 0.11 and 0.10, respectively. These heritability values were generally lower than or in the bottom range of the intervals presented for the semen traits in Table 8.

In contrast to our findings, the authors of [15] obtained higher values of the heritability coefficients for both basic and derived boar-semen characteristics. With the use of different model equations, the heritability coefficients presented in their study reached values of 0.58, 0.49, 0.38, and 0.42 for SV, SC, MO, and TNS, respectively. However, their study was designed with data from boars of nine breeds and ten groups of crossbreeds. In addition, the trait values were designed as arithmetic averages over all samplings for a given boar. The higher estimates obtained could be related to not only additive genetic variance but also a part of the permanent environment of the boar which could have a cumulative effect on the average values for each boar. Most studies focused on the estimation of genetic parameters with single- or multi-trait animal models. Ref. [35] estimated the genetic parameters of the total sperm count for three breeds bred on two farms using a random regression model. The authors found a gradual increase in heritability from approximately 33 weeks of age to 153 weeks of age ranging from 0.27 to 0.48, respectively.

**Table 8:** Average heritability coefficients for sperm characteristics of dam and sire breeds reviewed in different studies.

	Mean for Dam Breeds (n) ^6^	Range ^7^	Source	Mean for Sire Breeds (n) ^6^	Range ^7^	Source
SV ^1^	0.22 (6)	0.17–0.24	[30,32,33]	0.25 (5)	0.21–0.29	[6,12,32]
SC ^2^	0.21 (7)	0.18–0.25	[12,30,32,33]	0.21 (7)	0.05–0.34	[6,11,12,30,32]
MO ^3^	0.18 (11)	0.07–0.31	[12,14,30,32,33]	0.21 (12)	0.12–0.42	[6,12,14,30,32,36,37]
AB ^4^	0.30 (8)	0.15–0.39	[14,30,32,33]	0.24 (7)	0.16–0.35)	[14,30,32,36,37]
TNS ^5^	0.18 (5)	0.12–0.26	[12,14,33]	0.21 (7)	0.17–0.30	[6,12,14,32,36,37]

^1^ Semen volume; ^2^ sperm concentration; ^3^ motility; ^4^ abnormal sperm; ^5^ total number of sperm; ^6^ arithmetic mean for heritability coefficient (in parentheses is number of coefficients taken into account); ^7^ and minimum and maximum for heritability coefficient from investigated studies.

The genetic relationships between traits, which were expressed as correlation coefficients, reached low to intermediate values in both the positive and negative directions in our study. The strongest appeared to be a relationship between SV and SC (−0.701 and −0.580 for sire and dam boars, respectively). This negative relationship has also been well documented in other studies [11,32]. Li et al. [30] found a moderately negative correlation between SV and SC only for the Duroc breed, whereas for the Landrace and Yorkshire breeds, the correlation was close to zero. Generally, the estimated genetic correlations had values for sperm quality traits that were different from those reported in previous analyses [11,12,14,18,30,32,33]. The genetic correlation between libido and sperm motility had moderately positive values in our study (0.430 and 0.525 in the D and S datasets, respectively) and was close to zero (0.05 for Duroc) or moderately negative (−0.41 for the Yorkshire breed) according to the findings of [6]. When interpreting correlation coefficients with libido, the phenotypic expression of the trait could be considered. This trait is evaluated subjectively—usually according to a set scale that is similar to those for exterior traits. The scale was set so that the most desirable performance was associated with the highest value (5) in our study, but it was associated with the lowest value (1) in [12]. Generally, this genetic correlation revealed that selection for libido would bring the most advantageous improvements in motility and the proportion of abnormal spermatozoa and vice versa.

## 5. Conclusions

The effects of the insemination station and the year of collection were the main sources of the explained variability in the evaluated semen characteristics and libido of boars of both dam and sire breeds. The heritability of traits in both groups of breeds showed similar values, except for sperm concentration, which, in the case of dam breeds, had a significantly lower heritability. The genetic correlations between most of the semen characteristics and libido were found to be desirable, indicating that common selection would lead to joint improvement. Assessments are essential to ensure an effective genetic-evaluation system for boars in the context of economic consequences for AI stations and integrated production systems. We anticipate that the incorporation of genomic information on an individual basis can enhance the selection accuracy and, thus, selection progress of animals. Other studies should be oriented toward detecting some novel candidate genes for sperm characteristics and genomic evaluation of further traits that are included as selection criteria in boar populations.

## Figures and Tables

**Table 1:** Basic statistics of the reproductive traits of the evaluated boar datasets.

	Dataset D ^1^			Dataset S ^2^		
	*n* Ejaculates	Mean	s.d. ^3^	*n* Ejaculates	Mean	s.d. ^3^
SV ^4^ (mL)	32.333	271.29	107.28	24.468	250.60	97.93
SC ^5^ (10^−3^/mm^3^)	32.079	357.29	148.90	24.172	399.75	152.26
MO ^6^ (%)	32.188	77.16	6.56	24.320	76.95	7.46
AB ^7^ (%)	31.411	10.25	5.00	23.746	9.87	5.36
TNS ^8^	32.025	90.99	40.22	24.134	94.89	39.32
NFS ^9^	31.824	63.16	28.14	23.942	65.91	27.76
LI ^10^	32.384	3.62	1.22	24.505	3.61	1.17

^1^ Contains data from dam breeds; ^2^ contains data from sire breeds; ^3^ standard deviation; ^4^ semen volume; ^5^ sperm concentration; ^6^ motility; ^7^ abnormal sperm; ^8^ total number of sperm; ^9^ number of functional sperm; and ^10^ libido.

**Table 2:** Proportion of variance (in percent) explained by different effects on reproductive traits in boars of dam breeds (dataset D).

	SV ^1^	SC ^2^	MO ^3^	AB ^4^	TNS ^5^	NFS ^6^	LI ^7^
Month	1.82	0.36	0.07	0.07	0.67	0.62	0.20
Age	5.80	0.61	0.64	2.34	4.03	3.85	1.38
Interval	3.28	1.77	4.86	0.72	4.95	4.47	1.00
StatYear ^8^	14.18	37.95	55.80	22.33	27.08	26.72	22.77
Breed	1.17	0.22	0.66	0.04	0.20	0.06	2.73
Residual	73.75	59.09	37.98	74.49	63.08	64.28	71.93

^1^ Semen volume; ^2^ sperm concentration; ^3^ motility; ^4^ abnormal sperm; ^5^ total number of sperm; ^6^ number of functional sperm; ^7^ libido; and ^8^ combined effects of the insemination station and year of collection.

**Table 3:** Proportion of variance (in percent) explained by different effects on reproductive traits in boars of sire breeds (dataset S).

	SV ^1^	SC ^2^	MO ^3^	AB ^4^	TNS ^5^	NFS ^6^	LI ^7^
Month	1.69	0.98	0.18	0.24	1.65	1.72	0.22
Age	8.77	1.11	1.06	1.65	5.52	4.57	0.67
Interval	2.35	4.47	6.46	0.49	4.67	3.70	0.44
StatYear ^8^	16.63	20.28	52.73	12.89	16.25	16.88	17.00
Breed	3.31	0.50	0.54	0.04	1.50	1.25	0.31
Residual	67.25	72.67	39.02	84.69	70.40	71.87	81.36

^1^ Semen volume; ^2^ sperm concentration; ^3^ motility; ^4^ abnormal sperm; ^5^ total number of sperm; ^6^ number of functional sperm; ^7^ libido; and ^8^ combined effects of the insemination station and year of collection.

**Table 4:** Coefficients of heritability (on the diagonal) and the genetic (above the diagonal) and phenotypic (below the diagonal) correlations of reproductive traits in boars of dam breeds. Values in parentheses represent the standard errors of estimations.

	SV ^1^	SC ^2^	MO ^3^	AB ^4^	LI ^5^
SV ^1^	0.280 (0.018)	−0.580 (0.025)	−0.441 (0.035)	−0.147 (0.029)	0.459 (0.038)
SC ^2^	−0.332	0.099 (0.018)	0.396 (0.022)	−0.021 (0.020)	−0.268 (0.028)
MO ^3^	−0.004	0.037	0.141 (0.019)	−0.414 (0.031)	0.430 (0.031)
AB ^4^	0.052	0.066	0.105	0.237 (0.016)	−0.561 (0.023)
LI ^5^	0.134	−0.099	−0.015	−0.044	0.130 (0.017)

^1^ Semen volume; ^2^ sperm concentration; ^3^ motility; ^4^ abnormal sperm; and ^5^ libido.

**Table 5:** Coefficients of heritability (on the diagonal) and the genetic (above the diagonal) and phenotypic (below the diagonal) correlations of reproductive traits in boars of sire breeds. Values in parentheses represent the standard errors of estimations.

	SV ^1^	SC ^2^	MO ^3^	AB ^4^	LI ^5^
SV ^1^	0.259 (0.021)	−0.701 (0.42)	−0.241 (0.031)	0.245 (0.049)	0.401 (0.033)
SC ^2^	−0.323	0.276 (0.035)	0.550 (0.042)	−0.407 (0.38)	−0.507 (0.042)
MO ^3^	0.067	−0.055	0.109 (0.028)	−0.349 (0.038)	0.525 (0.034)
AB ^4^	0.083	0.017	0.051	0.220 (0.023)	−0.273 (0.044)
LI ^5^	0.170	−0.145	0.082	0.027	0.342 (0.036)

^1^ Semen volume; ^2^ sperm concentration; ^3^ motility; ^4^ abnormal sperm; and ^5^ libido.

**Table 6:** Genetic parameters for the total number of sperm and number of functional sperm in boars.

	Total Number of Sperm		Number of Functional Sperm	
	D ^1^	S ^2^	D ^1^	S ^2^
Additive genetic variance	175.3	153.2	91.4	96.2
Variance of permanent effects on boars	316.9	319.3	156.3	148.7
Residual variance	700.0	777.9	351.1	387.4

^1^ Dataset for dam breeds; ^2^ dataset for sire breeds.

**Table 7:** Basic statistics of the estimated breeding values and accuracies (in parentheses) for all evaluated breeding boars.

	n	Mean	Min.	Max.	s.d. ^1^
SV ^2^	1209	−2.63 (0.33)	−107.16 (0.01)	138.31(0.77)	28.70 (0.23)
SC ^3^	1209	2.51 (0.21)	−63.17 (0.01)	92.10 (0.56)	19.73 (0.14)
MO ^4^	1209	0.24 (0.26)	−3.64 (0.01)	4.10 (0.65)	0.84 (0.17)
AB ^5^	1209	−0.44 (0.30)	−5.48 (0.01	5.35 (0.73)	1.39 (0.20)
LI ^6^	1209	0.03 (0.24)	−0.87 (0.01)	0.91 (0.61)	0.19 (0.16)

^1^ Standard deviation; ^2^ semen volume; ^3^ sperm concentration; ^4^ motility; ^5^ abnormal sperm; and ^6^ libido.

## Data Availability

The derived data supporting this study are available from the corresponding authors upon reasonable request.

## Supplementary Materials

The following supporting information can be downloaded at: https://www.mdpi.com/article/10.3390/genes14112003/s1, Figure S1: Average monthly trends for the reproductive traits and datasets of boars; Figure S2: Effect of boar age in months on the reproductive traits and datasets of boars; Figure S3: Effect of intervals between two subsequent ejaculations on the reproductive traits and datasets of boars.
