# Supplementary material for: An Assessment of the Genetic Parameters of Boars’ Reproductive Traits
Source: Genes (Basel). 2023 Oct 27;14(11):2003. doi: 10.3390/genes14112003 (PMC10671126; doi:10.3390/genes14112003)
Supplement: Supplementary file 1 [file genes-14-02003-s001.zip › genes-2648896-supplementary.pdf]

genes

Supplementary material

An Assessment of the Genetic Parameters of Boars' Reproductive Traits

Emil Krupa[1]*, Zuzana Krupová[1], Eliška Žáková[1], Jiří Bauer[2], Nina Moravčíková[3], Irena Vrtková[4]

[1] Institute of Animal Science, 10400 Prague, Czech Republic; krupova.zuzana@vuzv.cz (Z.K.); zakova.eliska@vuzv.cz (E.Ž.)
[2] Czech-Moravian Breeders Association, 25209 Hradistko, Czech Republic; bauer@plemdat.cz
[3] Institute of Nutrition and Genomics, Slovak University of Agriculture, 94901 Nitra, Slovak Republic; nina.moravcikova@uniag.sk
[4] Department of Morphology, Physiology and Animal Genetics, Mendel University, Zemedelska 1, 61300 Brno, Czech Republic; irena.vrtkova@mendelu.cz
* Correspondence: krupa.emil@vuzv.cz

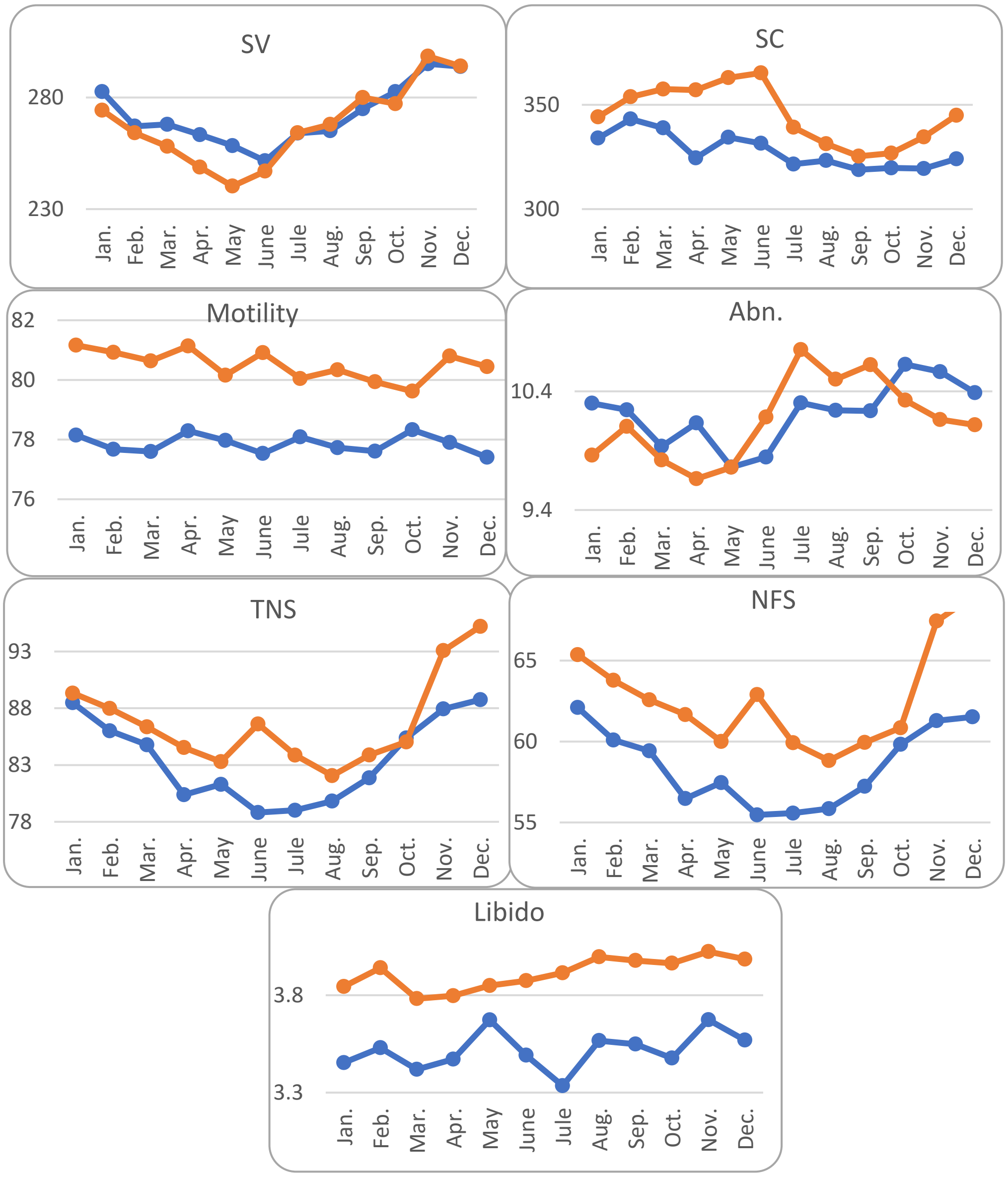

Figure S1: Average monthly trends for reproductive trait and dataset of boars (blue line = dataset D, orange line = dataset S)

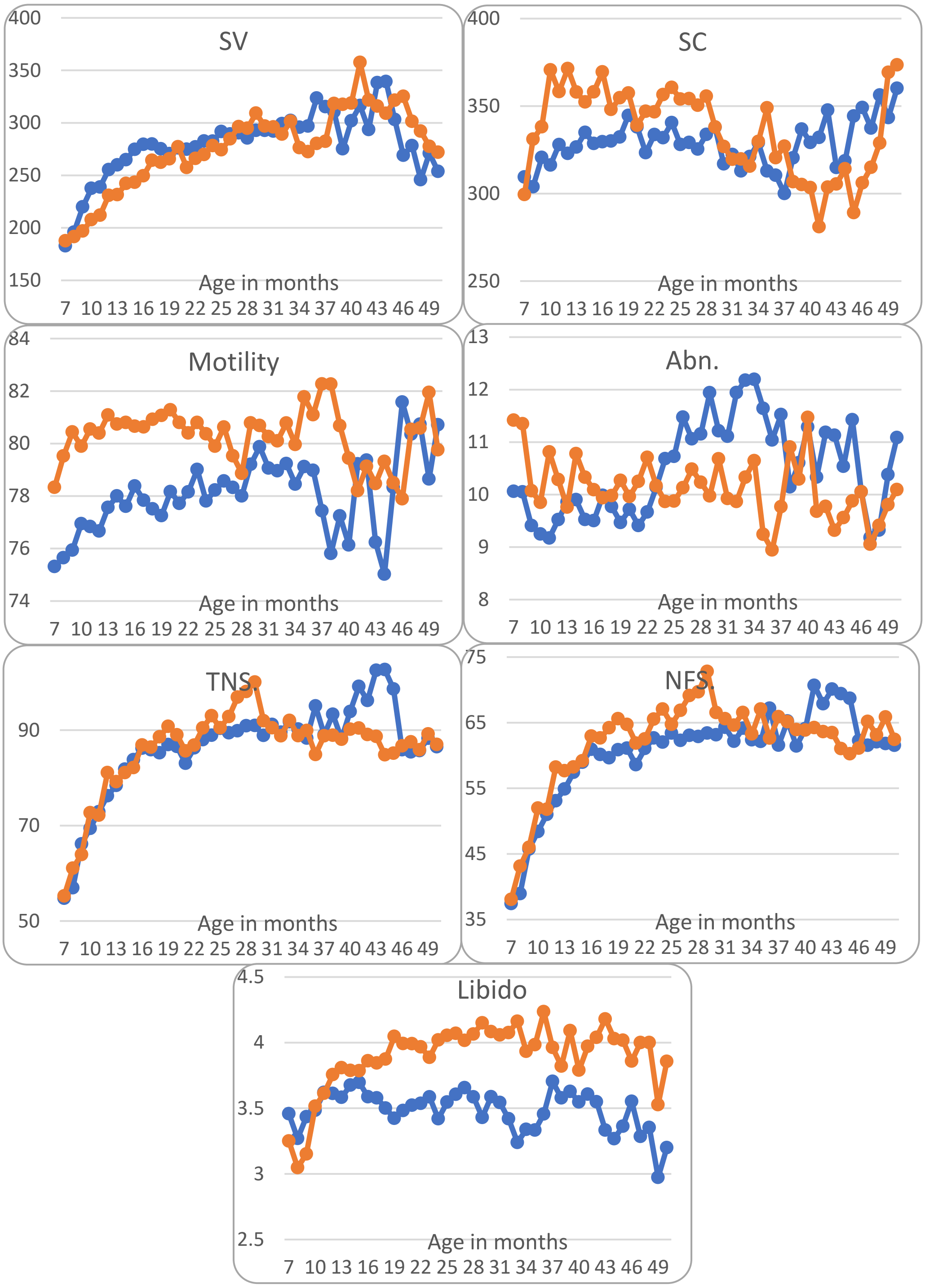

Figure S2: Effect of boar age in month on reproductive trait and dataset of boars (blue line = dataset D, orange line = dataset S)

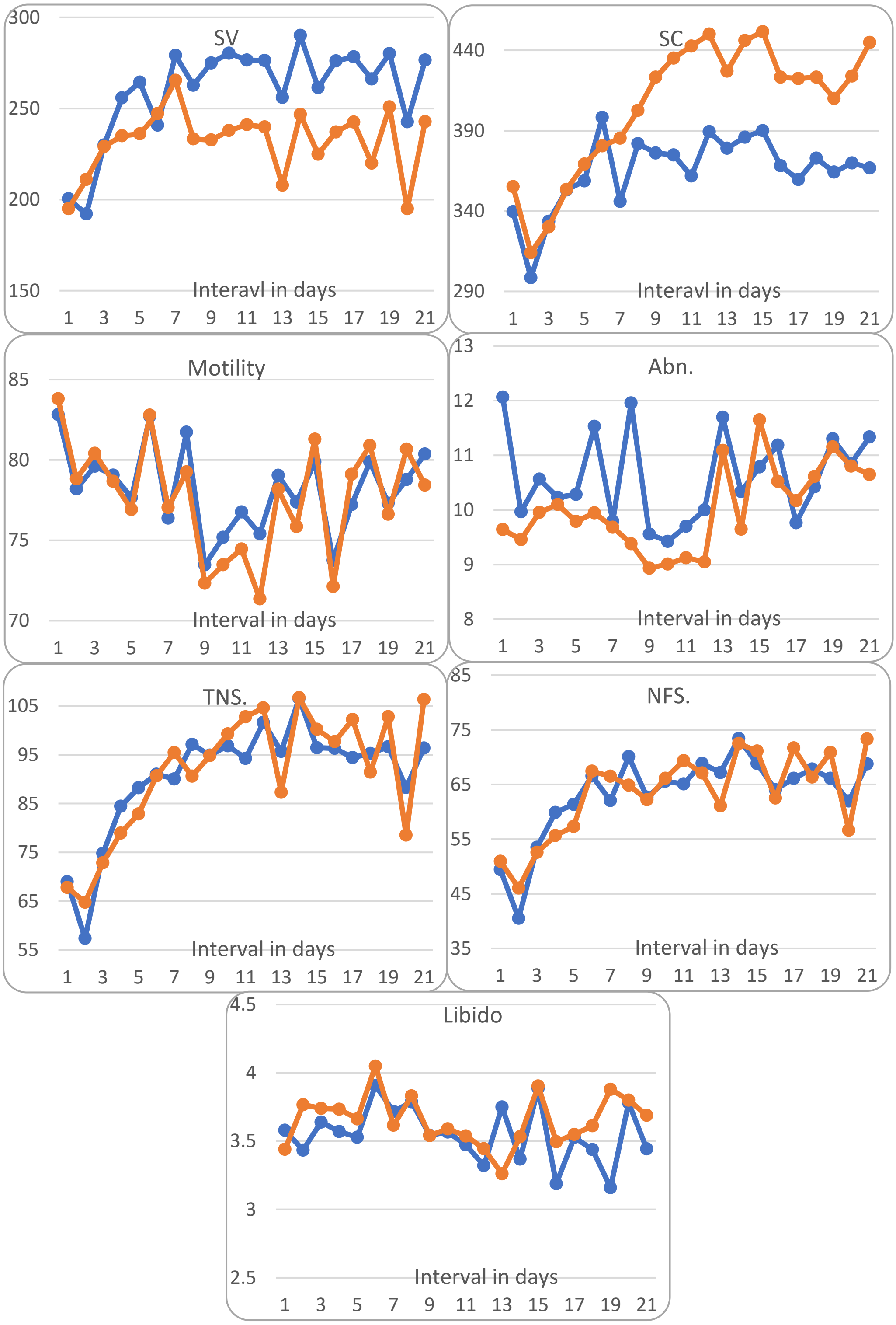

Figure S3: Effect of intervals between two subsequent ejaculates on reproductive trait and dataset of boars (blue line = dataset D, orange line = dataset S)
